# An exploration into the motivation for gluten avoidance in the absence of coeliac disease 

**Published:** 2018

**Authors:** Lucy Harper, Justine Bold

**Affiliations:** 1 *Freelance Nutritional Therapist, UK *; 2 *Allied Health and Social Sciences, University of Worcester, Worcester, WR2 6AJ, UK *

**Keywords:** Non-coeliac gluten sensitivity, Non-celiac wheat sensitivity, Gluten intolerance, Gluten-free, Coeliac disease, Self-management of symptoms

## Abstract

**Aim:**

To explore the motivation for gluten avoidance in the absence of coeliac disease (CD) and ascertain what symptoms are triggered by gluten and what beliefs/reasons influence this decision.

**Background:**

Links between physical/psychological symptoms and gluten in CD are well known but less is known about those who self-select a gluten-free diet (GFD) in the absence of CD.

**Methods:**

An empirical study using responses to an anonymous on-line questionnaire. Closed questions were used as a screening tool to exclude participants who had CD, wheat allergy or were following a low FODMAP diet. Data from participants using a GFD in the absence of a medical diagnosis was then analysed using thematic analysis.

**Results:**

120 initial responses, 87 were completed in full. 23 respondents fulfilled the inclusion criteria for thematic analysis. 7 different themes emerged, including one for signs/symptoms. Other themes identified included difficulties of a GFD, health beliefs, feelings and influence on decision to follow a GFD. Responses indicate that the reasons for gluten avoidance are in the most part reasoned and logical and were based around participants’ self-management of symptoms.

**Conclusion:**

Symptoms included those typical of irritable bowel syndrome (IBS), but also infertility, low mood/energy, immune function and weight management and visual and auditory hallucinations. It appears the majority of responses analysed thematically could fit into the spectrum of non-coeliac gluten sensitivity (NCGS). Findings also suggest more support at all levels of medical care may help patients establish if it is gluten, rather than wheat or FODMAPs particularly fructans that are contributing to signs/symptoms.

## Introduction

 There has been an exponential growth in the gluten-free (GF) foods market. This may be due to increasing consumer awareness of the contribution or *perceived* contribution of gluten to negative health beliefs and physical or psychological signs/symptoms. Since 2011, the gluten-free market has been increasing at 12.6% a year (1) but the number of people diagnosed with Coeliac Disease (CD), a chronic inflammatory disorder of the small bowel, requiring strict adherence to a gluten-free diet (GFD) to maintain health is not increasing at this rate (2). A UK population based study (3) found a fourfold increase in the incidence of CD over a 22-year period – even this however would not account for the growth in the GF market. 

 It has been suggested that the growth in the GF market could be down to people following ‘fad’ diets (4-6). Other possible contributory factors are the endorsement of GFD by celebrities and sportspeople (7) and of the publication of international bestselling books such as ‘Wheat Belly’ by Dr Davis (8) or ‘Grain Brain’ by Dr Perlmutter (9). It could be argued that with higher levels of internet usage, there is greater awareness of allergies and intolerances, and so a greater awareness of the benefits of reducing or avoiding gluten to those with inexplicable symptoms (10,11). 

Despite CD being a well-studied condition, for every diagnosed person it is estimated that there are eight undiagnosed cases (12). One of the reasons for this shortfall in diagnosis is the wide range of presentations from gastroenterological to neurological symptoms, and to the similarity in some signs/symptoms to those of irritable bowel syndrome (IBS) (13). Although non-coeliac gluten sensitivity (NCGS) is a condition which its existence is still debated, it has begun to be accepted as a clinical entity (14, 15) despite the absence of diagnostic markers (16). A 2014 report puts the figure of 10% of the population having a gluten related disorder (17). NCGS may include abdominal symptoms but also systemic manifestations such as dermatitis, tiredness, aches and pains (15). A few papers are now emerging linking NCGS with neuropsychiatric manifestations as the prime presentation (18) as well as linking it to autism, schizophrenic, ataxia, hallucinations (19) and depression (20) although this remains controversial due to conflicting research results (16). Research is beginning to show that NCGS may play a major role in irritable bowel syndrome (IBS) (12, 21, 22) with a recent study indicating that adherence to a GFD alleviated symptoms and improved quality of life in 34% of patients with diarrhoea-dominant and mixed type IBS (23). 

Issues with low fermentable oligosaccharides, disaccharides, monosaccharides, and polyols, otherwise known as FODMAPs, which are naturally high in all gluten foods may also be contributing to the rise in the number of people avoiding gluten and leading to claims by some researchers that it is not gluten *per se* causing symptoms but the fermentation of FODMAPs with consequent gastrointestinal discomfort (24). 

Another possible factor in the rise in GF diets is the increase in autoimmune diseases which is a pathophysiological state in which the immune system attacks the body’s own tissue (25). Certain autoimmune conditions such as thyroid disease, predominately Hashimoto’s but also Graves, and Type I diabetes have been linked to gluten as being a possible trigger (26-28).

## Methods

An empirical study using an on-line questionnaire incorporating qualitative thematic analysis was selected as being an appropriate methodology in achieving the objective of gathering respondents’ experiences on the basis of it being well suited to exploring a range of perspectives (29-31). This method of exploratory research has been shown to lead to better understanding and to improvements in health services (32). By using open-ended questions, participants can express themselves without the restraints that would otherwise be imposed upon them by the standard closed question or multiple-choice questionnaire (32). Qualitative thematic analysis is recognised for its’ flexibility in how it can be used and potential to provide rich and detailed data (33). At present this type of data appears to be lacking in the area under study. 

An on-line survey was selected to access a wider range of views given the specific population group required and the financial constraints (as postgraduate research without funding) and for its anonymity which is ideal for sensitive data collecting and as requiring less of the participant in terms of time compared to either an interview or a focus group (34-36). 

Ethical approval was obtained through the University of Worcester, UK. Data was collected through an electronic anonymous questionnaire using Survey Monkey over the period of one month in 2015. The questionnaire was launched via social media using the University course Facebook and Twitter pages and by sending links to professional associations in the UK. An estimated 9000 individuals were reached by this means. The online survey was open to all those who avoid gluten in the absence of CD. A short questionnaire was devised and piloted on 6 participants known to the researchers following which minor changes to the questionnaire were introduced. Closed questions were used as both a screening tool and to help establish certain facts / criteria which would support the data obtained for use in data analysis. 

The criteria for data to be selected for thematic analysis was that the respondent had tested negative for CD, had been consuming gluten at the time of the test and for whom FODMAPs did not appear as the sole reason for their symptoms. If responses indicated a wheat allergy the responses were only used in the statistical analysis not in the thematic analysis. The remaining responses were then analysed manually using thematic analysis. This involved systematic coding and the finding of categories and themes or patterns and a degree of interpretation (31,33).

The use of an on-line survey considerably reduces the issues of having to transcribe audio recordings or what might be illegible handwriting reducing likelihood of errors in analysis (34,35). To reduce the risk of systematic error, the second researcher checked the lead researchers’ interpretations of the data for thematic analysis, as it is recognised that different researchers may have varying interpretations of the same data. This practice is often referred to as member checking (31). 

Participants in this study were informed as to the purpose of the research, who was undertaking the study, how the data would be used and what was being asked of them. They were also assured of anonymity and that no data would be used that could identify them. Sign-posting was also provided for support and e-mail addresses were provided should respondents have wished to contact the researchers about the study. 

**Figure 1:**
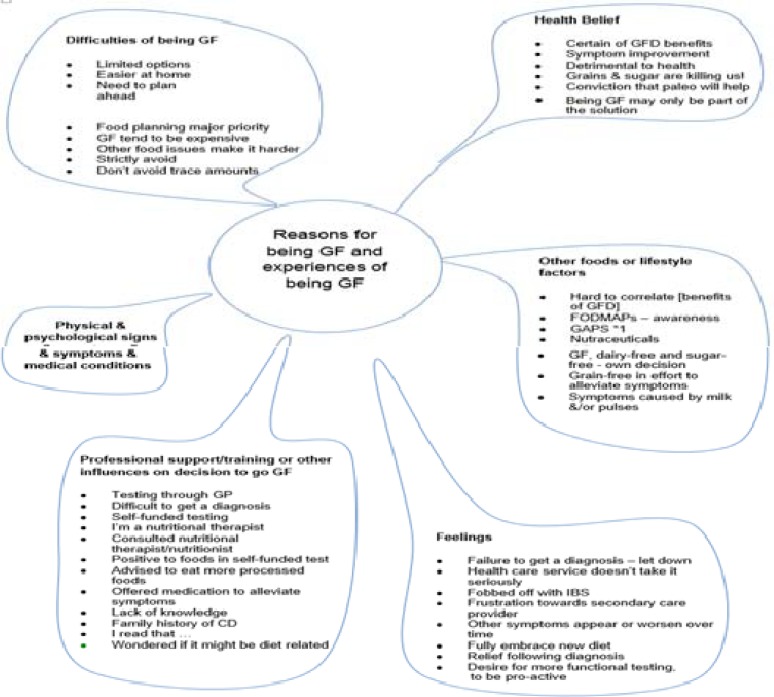
Thematic map; Summary of the themes from the data analysis

**Table 1.** Illustrating responses to closed questions. This table indicates the number of respondents who answered the closed questions in the affirmative:

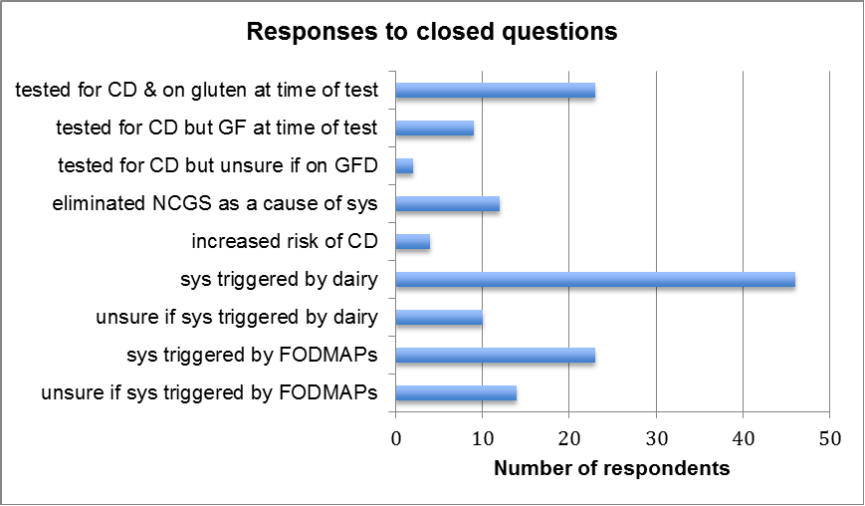

**Table 2:** Signs, symptoms and general feelings said to improve on a GFD

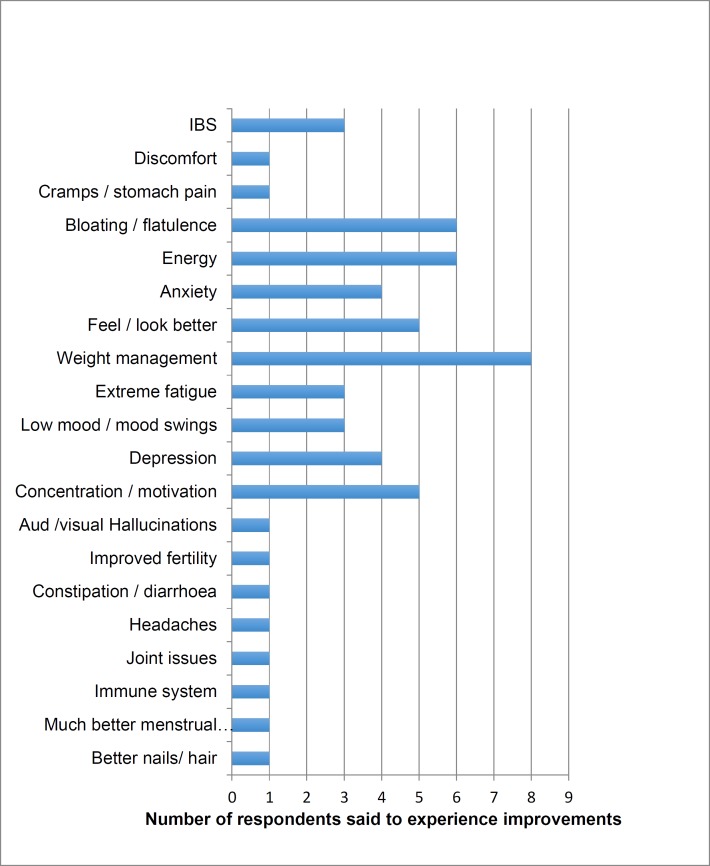

## Results

There were 120 responses to the questionnaire including 6 participants involved in the pilot study. It was completed in its entirety by 72.5 % (n=87) of respondents. Content analysis was used as a screening tool for the responses to be analysed thematically. The nominal data obtained from the closed questions was analysed. The data obtained from the open question from the 23 respondents who fulfilled the inclusion criteria was analysed thematically. Some of the responses were very brief and did not complete the open question. These uncompleted questionnaires were excluded from any further data analysis. 

The responses to the closed questions are illustrated in Table 1. The analysis of data from the open question inviting participants to write about their reasons for and experience of going GF initially generated 121 codes, which after putting all signs/symptom-related codes under one single code, were condensed down to 50 codes and categorised further into different themes. Six themes emerged (see Table 2 for symptoms experienced by respondents included in thematic analysis and Figure 1 Thematic Map). 

**Physical and psychological signs and symptom**

This theme elicited the most information associated with the intake of gluten foods. IBS-type symptoms were mentioned most frequently at 56.5% (n13) followed by 21.7% (n5) mentioning fatigue and the same number referring to improvements in energy on a GFD. Negative feelings of emotion attributed to gluten were mentioned by 7 (30%) respondents, including depression by 4 respondents (17.4%) and sadness by another, whilst 3 claimed a GFD resulted in lower anxiety. Gluten was said to effect cognitive function by 5 (21.7%) of the respondents indicating that it negatively impacted thinking and concentration, created ‘brain fog’ and made them just want to sleep. The implications of the degree to which gluten could affect someone and the ramifications of an undiagnosed issue was clearly brought home by this mother writing about her daughter’s rapid health improvements once gluten was eliminated from her diet affecting academic work and motor skills:

*“…Very quickly within 4-6 weeks, her teachers at school commented on what a remarkable turnaround her concentration is now, she is responsive, wants to join in class discussions and she has even gone up a set in Maths! Her writing has improved massively from unrecognizable to now being able to concentrate to write many paragraphs in creative writing…”*

Whilst it was not always possible to establish a causal link to weight loss on a GFD as there may have been a combination of factors such as the elimination of all grains or sugar as in the case of respondent (n17). Out of the 5 respondents who changed to a grain-free diet only one stated weight loss which was a surprising given that it is precisely the grain-free group rather than just GF group that one would have assumed would lose the most weight. Of the other 6 respondents who lost weight on a GFD, all attributed it to going GF. Respondent (n18) correlated a three kilo weight gain following intake of just one slice of bread, which cannot be explained in terms of calorie intake.

In 7 cases (30.5%) a GFD had been adopted to alleviate symptoms related to autoimmune disorders and other 

 medically diagnosed conditions. Most frequently referred to was thyroiditis, mostly Hashimoto’s but also Graves, of the five respondents who mentioned thyroiditis, two claimed a GFD reversed their condition and two that it led to significant improvements: 

*“…I tried taking gluten out completely & this time 100% including when eating out - checking thoroughly. Within a month my thyroid hormones were back within normal ranges… I still avoid gluten and plan to for life. This is because I have now seen a direct correlation between eating gluten and my thyroid health”* (29).

Another respondent mentioned that they “…avoid gluten mainly to avoid bloating, stomach pains and extreme fatigue due to multiple sclerosis…” the implication here is that a GFD helps alleviate fatigue. 

Feeling better or healthier or looking better was reported by 6 (26%) of respondents with skin problems being another area said to improve on a GFD; with 4 (17.4%) mentioning either skin colouring, roughness, general improvements, less acne and improvements in rosacea. Other symptoms said to improve by at least one GF respondent included: congestion, headaches, constipation, joint issues, immune function, condition of hair and nails.

An area not often alluded to in research was that of improved fertility on a GFD with one respondent mentioning she “… *actually managed to not just get pregnant but carry a pregnancy to viability for the first time, I had previously lost each pregnancy and had difficulty getting pregnant…”* and another claiming *“…much, much less pain and bloating associated with menstruation,…”*.

Another rarely reported presentation attributed to gluten was that of a cessation in visual and auditory hallucinations. 

*“After 6 weeks of a strict gluten and dairy free diet and focusing on gut repair I stopped getting any form of hallucinations [sic] or voices in my head…” *(C) 

See Table 2 for 23 respondents signs and symptoms as included in the thematic analysis. See Figure 1 for thematic map.

**Health Beliefs**

Most academics consider health beliefs from a point of view of ill health. However, in this paper health beliefs are considered in two separate categories: first, as beliefs that stem from an anecdotal based approach to health such as a belief that all bread is fattening therefore avoided and a second category of ‘self-evidenced’ belief. At the most, two respondents appear to have eliminated gluten under the first criteria of an anecdotal based approach; with one hypothyroid and “B12” chronically deficient respondent convinced that a Paleo diet would be beneficial whilst another respondent made the statement that “Grains and sugar are slowly killing us all!!’. This respondent also stated that being grain-free had alleviated anxiety, depression, acne and rosacea therefore it could be said that this health belief could have fallen into the second category of ‘self-evidenced health belief’ held because of physical or psychological symptoms. Three respondents made a very clear link with the re-introduction of gluten and the return of signs/symptoms whilst another respondent stated that the link was not immediately obvious. Over a quarter (n6) expressed that they simply felt better, felt healthier or looked better. 

Professional support or training or other influences on the decision to go GF was another theme to emerge. Only 1 respondent mentioned professional support from a Doctor (General Practitioner) whilst two respondents mentioned seeking support from Nutritional Therapy Practitioners, with one commenting that this was following the inadequate support offered in secondary care in the UK. The disappointment with medical advice or the lack of access to dietary advice was implied by a couple of respondents which led one to reject medication in favour of gluten elimination which they report led to them feeling better and losing weight. Some mentioned ‘wondering’ if symptoms might be diet related or ‘reading’ or ‘hearing’ that gluten could contribute to symptoms and leading them to adopt a GFD.

**Feelings: **This theme covered the difficulty and delay in getting a diagnosis, the feelings of frustration and suffering endured as a consequence but also the feelings of relief following a possible resolution. Only one respondent claimed feeling that their symptoms were not taken seriously, they appeared to resent a diagnosis of IBS. One respondent tried to go back to eating “normally” which raised the question as to whether they see themselves as not being “normal” although they had been on a GFD for three years and it helped them “feel better”. Whilst some appeared reluctant to adhere to a GFD others fully embrace it. The desire to take responsibility and to be pro-active with regards to understanding their own and their families’ health was voiced by a respondent who felt that taking charge of their health was empowering and wished they had the financial means to do more private tests. Another respondent felt that their issues were multi-factorial and indicated a struggle with accessing support.

**Difficulties of adhering to a GFD**

This was a theme that overlapped with that surrounding feelings with a respondent mentioning the psychological difficulties made harder still when accompanied by other dietary requirements and more burdensome when young children were involved. Other difficulties were mentioned by three respondents and included the difficulties of avoiding gluten especially when outside the home environment, the need to be very organized and the cost of GF foods. Food planning became a major priority. The difficulties encountered when out and about appeared to depend not only on confidence with foods but also on how strictly respondents avoid gluten.

**Other foods and lifestyle factors**

Under this theme is the recognition by some that it was not always easy to associate symptom improvement to a GFD. Three respondents acknowledged that other factors such as certain FODMAPs foods such as pulses or dairy, usually milk, may be contributing to a degree to some symptoms.

## Discussion

The data analysis was of a small sample and it is acknowledged that despite meeting the inclusion criteria there is a possibility that a number of participants have undiagnosed CD. It is also acknowledged that the method used may have restricted responses to certain groups such as to those who are comfortable with internet usage, of a particular education level and interested in health issues as the main posting to the survey was via sites with an interest in nutrition which could have led to greater homogeneity and therefore sampling bias (36-38). It is believed that all the respondents were UK based. Hence, whilst this convenience sample is not necessarily a representative sample and the study is not generalizable it was felt that it would contribute to greater understanding of the issues around gluten avoidance (31). 

Other limitations relate to the use of an online survey. Answers may be limited due to a type of device – for instance if the survey is being viewed on a small device such as an iPhone, the length of a reply to an open-question could be shorter than a reply posted via a computer (39). 

The uncontrollability of a survey on social media also made it impossible to gauge response rates. Due to the unpredictability in the volume of responses33, and in this case the higher number than anticipated, in addition to the financial and time constraints, on advice from academics at the University, the data obtained from the closed questions was used for statistical analysis and also served as a screening tool for the data that would be used in the thematic analysis. The criteria for data to be selected for thematic analysis was that the respondent had tested negative for CD, had been consuming gluten at the time of the test and for whom FODMAPs or wheat allergy did not appear as the sole reason for their symptoms. 

Whilst the categorisation of signs and symptoms may appear straight forward certain categorisations were found to require extra consideration and care such as in this descriptive comment made by one respondent: “gluten based products make me slow, tired, fat, lazy, heavy, sluggish and grumpy …It’s not natural”. As all data was obtained through the anonymous questionnaire it was not possible to go back to the respondents in order to have any statements further explained. 

Comparing results of one study to another is difficult as the terminology as well as methodology can vary considerably. For instance, some people may mention IBS type symptoms whilst other may specify individual IBS symptoms. This can also impact on thematic analysis as care has to be taken not to double count data. Another example of differing terminology is that surrounding feelings. Some may use the term depression when referring to low-mood or vice versa. Whilst this survey indicated gluten being associated with depression more than with anxiety as in the Peters *et a.l* (20) study, the Aziz *et al.* (2014) (40) population-based survey indicated much higher rates of anxiety compared to depression (21% compared to 13%).

Whist participants were asked to confirm if they reacted to common UK FODMAPs foods such as apples, pears, onions, leeks and pulses out of the 23 whose data was thematically analysed 6 reported reacting to these foods. They were included in the analysis because it was felt that their symptoms did not appear to be only down to a FODMAPs sensitivity as they were reporting symptoms not associated with FODMAPs at the time the research was undertaken. A recent study looking at the results from ten double-blind placebo-controlled (DBPC) studies involving a gluten challenge with 1312 adults concluded that 80% of patients with suspected NCGS could not be definitively classified as NCGS. One of the reasons for this is the contribution made to symptoms by FODMAPs and in particular fructans, and also the personal differences in gluten tolerance levels (41). This survey indicated that out of the 87 responders, including those whose data were used for thematic analysis, 53% claimed to have an issue with dairy, which compares to the 52.9% of respondents who avoid dairy in the population based survey on wheat avoiders (42); but differs substantially compared to the Aziz *et al.* (2014) (40) survey which only indicated a 3.9% dairy intolerance. Both the Aziz and Golley studies included over a thousand respondents although the studies were carried out in different countries. Difference could also be down to survey design. 

The improvements in auto-immune conditions following the adherence to a GFD are unsurprising when one considers the research carried out by Dr Wentz (2016) (43), a thyroid specialist, on her patients, 93% of whom say they feel better on a GFD. A retrospective study found that the prevalence of auto-immune disease, primarily Hashimoto’s, is the same in those with NCWS as in those with CD (28).

This study revealed some more unusual symptoms related to gluten in the absence of CD, which are not usually measured during quantitative studies. These include the auditory and visual hallucinations triggered by gluten as mentioned by one respondent who reported that following a strict GF and dairy-free diet led to complete cessation of hallucinations. It has been shown that there is a much higher prevalence of positive antigliadin antibodies amongst those suffering neurological symptoms of unknown cause – 57% compared to 12% in healthy patients (44). In this survey, no other neurological symptoms were mentioned apart from hallucinations, depression and anxiety. However, the prevalence could be higher than is revealed here due to stigma attached to mental health (45), or simply because neurological symptoms are not associated with a food intolerance (18). 

Another less recognised association with gluten intolerance was that of improved fertility following the introduction of a GFD. It has been shown that amongst women with unexplained infertility, there is a significantly higher proportion with CD, with one study indicating a prevalence rate of 5.9% and of IBS affecting over 50% (46,47). A case report has been published on the possible association of NCGS as a possible factor in unexplained infertility (48). Research on women presenting with endometriosis have also shown a higher prevalence rate of 2.5% testing positive to CD (49). 

**Recommendations**

The responses from the survey indicated that the participants’ reasons for gluten avoidance in the absence of a medical diagnosis of CD were for the most part reasoned and logical. The vast majority of participants believed that adhering to a GFD led to improvements in signs and symptoms. The growth in the gluten-free market (1), the identification of symptom alleviation as a factor in those who chose to adhere to a GFD as could be extrapolated from this study, and mounting evidence that CD is not the sole reason for gluten related symptoms (12-23) indicates that there is evidence to support testing of a GFD in clinical practice and for a NCGS diagnosis to be considered. Furthermore, there is a need for dietary guidance on a GFD from dieticians or nutrition practitioners. 

It appears that there is also a need for additional professional support to help establish if it is gluten *per se*, wheat or FODMAPs that are the cause of symptoms. In the UK, the National Institute for Health Care Excellence (NICE) (50) makes no mention of gluten sensitivity or intolerance in its nationally recognised pathways except in reference to CD although mention is now made with regards to removing single food items or high FODMAPs foods for those with IBS (51). Concerns with self-diagnosing a gluten issue but not having a CD test is that undiagnosed CD, and therefore, a more relaxed attitude towards avoiding all gluten, increases the risk of auto-immune diseases, bone disease, low fertility and malignancy in the form of cancer of the small bowel and oesophageal cancer (52). There is a need for greater public and professional awareness and understanding that a negative serological test or small bowel biopsy for CD does not mean that gluten can be consumed with impunity, especially if there are unexplained symptoms such as those mentioned in this research (53,54). 

Whilst it is recognised that health services generally have limited resources and doctors/general practitioners are often not in a position to support patients nutritionally, greater recognition of dietary means of managing symptoms should be encouraged. Patient care would benefit from greater inter-professional understanding and in the UK a wider use of Complimentary and Natural Healthcare Council (CNHC) (55) registered nutritional practitioners to alleviate the burden on nationalised healthcare. More research is also warranted into NCGS, especially the link between NCGS, IBS and FODMAPs.

## Conflict of interests

The authors declare that they have no conflict of interest.
